# Clinical Features and Survival Analysis of Lupus Nephritis among Patients with Systemic Lupus Erythematosus: A Three-Decade-Long Retrospective Cohort Study

**DOI:** 10.3390/biomedicines12092117

**Published:** 2024-09-18

**Authors:** Bianka Perge, Gábor Papp, Bernadett Bói, Nikolett Nagy, Eszter Gáspár-Kiss, Tünde Tarr

**Affiliations:** 1 Division of Clinical Immunology, Institute of Internal Medicine, Faculty of Medicine, University of Debrecen, H-4032 Debrecen, Hungary; pergebianka@med.unideb.hu (B.P.); papp.gabor@med.unideb.hu (G.P.); nagy.nikolett@med.unideb.hu (N.N.); gaspar.kiss.eszter@med.unideb.hu (E.G.-K.); 2 Department of Public Health and Epidemiology, Faculty of Medicine, University of Debrecen, H-4028 Debrecen, Hungary; boi.bernadett@med.unideb.hu

**Keywords:** SLE, lupus nephritis, survival, remission, low disease activity

## Abstract

**Background/Objectives:** Lupus nephritis (LN) is one of the most severe organ manifestations of systemic lupus erythematosus (SLE). The aim of our retrospective cohort study was to compare the clinical characteristics, therapy, survival, causes of death, and prognostic factors of LN and non-LN lupus patients. Moreover, we compared a wide spectrum of clinical data of LN patients diagnosed before and since 2005 to determine any changes in disease course and outcomes. **Methods:** We assessed the clinical and laboratory data of 384 SLE patients, out of whom, 127 patients were diagnosed with LN between 1990 and 2020. **Results:** Based on our observations, discoid LE, subacute cutaneous LE, antiphospholipid syndrome, Sjögren’s syndrome, and rheumatoid arthritis were more common in non-LN patients, while anemia and anti-RNP positivity were more frequent in LN patients. Development of LN did not affect survival rates; male sex and presence of APS were negative prognostic parameters in the non-LN group while achieving remission was a positive prognostic factor in both groups. Death caused by sepsis was more prevalent in the LN group. Serositis and neurological manifestations occurred less frequently in LN patients diagnosed after 2005. The use of mycophenolate mofetil became more common, and the cumulative corticosteroid dose decreased. The SLICC Damage Index score also decreased. **Conclusions:** Our study demonstrated that the disease course has changed in recent years, and the main therapeutic goal in both SLE and lupus nephritis should be to achieve remission because this significantly improves long-term prognosis and patient survival.

## 1. Introduction

Systemic lupus erythematosus (SLE) is a chronic systemic autoimmune disease that affects multiple organs. The disease is characterized by the loss of self-tolerance with the activation of autoreactive T and B cells and the production of pathogenic autoantibodies against self-antigens. One of the most important steps in pathogenesis is the formation of immunocomplexes and their deposition in various tissues and organs. Immunocomplex deposition in kidney glomeruli is associated with lupus nephritis (LN), which is considered one of the most severe organ manifestations [1]. The prevalence of LN among SLE patients is about 35%, but according to literature data, it ranges from 12–69% in different populations. It is more common and more severe in Asian, African, and Hispanic SLE patients [2]. The development of end-stage renal failure is more common in SLE patients with kidney involvement, and the mortality rate is 5–8 times higher than in the average population [3,4,5]. LN usually appears in the first five years of SLE but may develop later. However, it can also be the first manifestation of SLE [1]. Several workgroups have confirmed that LN is more common in male patients and juvenile SLE, as our workgroup also found [6,7,8,9,10,11]. The clinical presentation is heterogeneous, it can be a subclinical form with only laboratory abnormalities, but it can also appear in the form of nephrosis, nephritis, or acute kidney failure [1]. Renal biopsy is the cornerstone of LN diagnosis, which is also taken into account by the EULAR/ACR classification criteria for SLE. ANA positivity and Biopsy-proven class III or IV LN are sufficient to establish the diagnosis of SLE. The histological type of LN, the activity score, and the damage index can be determined with histological examination. Renal biopsy helps exclude other renal diseases or may confirm, for example, the co-occurrence of associated thrombotic microangiopathy (TMA) [12,13]. Renal biopsy is recommended in all SLE patients with persistent proteinuria exceeding 0.5 g per day, especially if this is accompanied by hematuria and/or the presence of cellular cylinders in the urine sediment. It should also be considered in cases where active urine sediment or chronic renal failure develops and this cannot be explained by other reasons [13]. The histological classification is based on the ISN/RPS 2003 classification of LN, which was updated in 2018 [14,15]. The histological type determines the course of treatment, and can also indicate the prognosis of the disease. There are several international guidelines that help in the induction and maintenance treatment of lupus nephritis [13,16,17]. In the last decade, the use of mycophenolate mofetil (MMF) has become more and more common in both induction and maintenance treatments. Targeted therapeutic options have also appeared. Belimumab became a registered drug for the treatment of LN and rituximab can also be used in severe cases [18,19]. Voclosporin, which is a novel calcineurin inhibitor (CNI), has also been approved for treating LN, and several other targeted therapies are under development [20,21,22]. Thanks to all of this, the therapeutic options for LN patients are constantly improving. Significant therapeutic advances have also been made in non-renal SLE. New effective guidelines help the treatments, additionally, a new targeted therapy, anifrolumab, has also been approved for the treatment of SLE [17,23,24]. The aim of treatment is low disease activity or remission [25,26]. The main therapeutic goal is to achieve remission and to minimize the corticosteroid dose or completely stop using corticosteroids. This way chronic organ damage may be decreased [27,28,29].

Several working groups consider lupus nephritis to be a negative prognostic factor in lupus [30]. However, the diagnostic and therapeutic options have also changed in recent decades; therefore, our objective was to examine the occurrence and characteristics of LN among SLE patients who were diagnosed after 1990 and received a regular follow-up since then at one of the largest Hungarian autoimmune centers. We compared the clinical characteristics, therapy, survival, causes of death, and prognostic factors of LN and non-LN lupus patients. Furthermore, in order to shed light on the achievements of the last decades, we compared a wide spectrum of clinical data as well as treatment modalities of LN patients diagnosed before and since 2005 and determined any changes in disease course and outcomes.

## 2. Methodology

### 2.1. Study Population

In our retrospective cohort study, we assessed the data of 384 Hungarian patients who were diagnosed with SLE between 1990 and 2020 and followed up regularly at the Division of Clinical Immunology (Faculty of Medicine, University of Debrecen), which is one of the largest tertiary referral centers in Hungary for systemic autoimmune diseases.

The diagnosis of SLE was established on the American College of Rheumatology (ACR) classification criteria (1982 and 1997) or SLICC (2012) criteria, according to the date of diagnosis [31,32,33]. Patients diagnosed with SLE before 2012 were revised according to the SLICC criteria for SLE; additionally, all SLE patients enrolled in the present study fulfilled the EULAR/ACR 2019 classification criteria for lupus [34]. The diagnosis of APS was based on the Sapporo (1999) or Sydney criteria (2006); patients diagnosed with APS before 2006 were revised according to the Sydney criteria [35,36]; moreover, they all met the criteria of the 2023 ACR/EULAR APS classification criteria as well [37]. The study was conducted in accordance with the Declaration of Helsinki, and approved by the Ethics Committee of our University (protocol number: 4879-2017) and the Policy Administration Services of Public Health of the Government Office (protocol number: 1660-4/2018).

### 2.2. Clinical and Laboratory Evaluation

All patients included in the study received therapy and regular follow-up care at our autoimmune center throughout the studied period, no patients were excluded due to missing data or lost to follow-up. Their medical records contained detailed information on medical history and treatments as well as clinical symptoms, physical conditions, and laboratory and other findings of each visit. The following demographic and clinical data were analyzed: sex, age, age at diagnosis, disease duration, histological class of lupus nephritis, clinical symptoms and laboratory results, immunoserological abnormalities, and applied treatments during the disease course. All LN patients underwent renal biopsies, which were performed at the Department of Nephrology (Institute of Internal Medicine, Faculty of Medicine, University of Debrecen), and the kidney biopsy samples were evaluated at the Department of Pathology (Faculty of Medicine, University of Debrecen). We used the current WHO classification system, ISN/RPS2003, ISN/RPS2018 for histological description [14,15,38]. Chronic renal failure was defined as GFR < 60 for at least 3 consecutive months and no improvement thereafter [39]. End-stage renal disease (ESRD) was defined when renal dialysis or renal transplantation was required. We determined the chronic organ damage of SLE with the SLICC Damage Index (SDI) [40]. The definitions of low disease activity and remission were based on international recommendations [25,26].

Immune serological parameters were determined from serum samples. The presence of antinuclear antibodies (ANA) and antineutrophil cytoplasmic antibodies (ANCA) was detected by an indirect immunofluorescence method. Enzyme-linked immunosorbent assay (ELISA) was used for the detection of the following antibodies: anti-dsDNA, anti-SS-A, anti-SS-B, anti-RNP, and anti-Sm, as well as antiphospholipid antibodies including anti-cardiolipin (aCL) IgG/IgA/IgM and anti-ß2GPI IgG/IgA/IgM antibodies. All laboratory measurements, including routine laboratory tests such as whole blood count, ions, kidney-and-liver function, and lipid panel, were performed at the Department of Laboratory Medicine, Faculty of Medicine, University of Debrecen.

### 2.3. Statistical Analysis

Statistical analysis was performed using SPSS Statistics for Windows, Version 28.0 (IBM Corporation, Armonk, NY, USA) and GraphPad Prism version 9.5 for Windows (GraphPad Software, San Diego, CA, USA). Values are expressed as mean and standard deviation (SD) or median with interquartile range (IQR) for continuous variables, and frequency with percentage for categorical variables. Continuous variables were compared with parametric Student’s *t*-test for two samples or with the nonparametric Mann–Whitney U test. Categorical variables were compared with Pearson’s chi-squared test or Fisher’s exact test. Kaplan–Meier analysis was used to estimate survival from the diagnosis and log-rank test was used to compare the survival curves. Significant variables on log-rank tests were further evaluated with univariate Cox proportional hazards regression models with estimation of hazard ratios (HR). Receiver operating characteristic (ROC) curve analysis was used to determine the optimal cut-off value for SDI as a prognostic factor for mortality. All statistical tests were two-sided, differences were considered statistically significant at <0.05 level and reported using *p*-values and/or 95% confidence intervals (95% CI).

## 3. Results

### 3.1. Demographic Characteristics

The population of our retrospective study consisted of 384 Hungarian SLE patients, of whom 339 (88.3%) were women and 45 (11.7%) were men. Their mean age at the time of data collection was 50.8 ± 13.4 years, their age at the time of SLE diagnosis was 33.3 ± 11.9 years, and the average duration of disease was 17.5 ± 8.0 years. Among the examined patients, 127 (33.1%) had lupus nephritis, while 257 patients (69.9%) did not have LN. Table 1 shows the demographic data of the patients.

Patients with LN were significantly younger at the time of both the SLE diagnosis and the data collection. The women-to-men ratio was the same in both groups; of note, LN was not more prevalent in male patients.

### 3.2. Clinical Characteristics and Laboratory Findings of LN and Non-LN Patients

We compared the clinical characteristics and laboratory findings of LN and non-LN patients (Table 2).

Among the clinical symptoms and associated diseases, subacute cutaneous lupus erythematosus (SCLE), discoid lupus erythematosus (DLE), secondary APS, Sjögren’s syndrome, and rheumatoid arthritis were significantly more common in the non-LN group. Regarding laboratory results, LN patients were affected by anemia and anti-RNP positivity significantly more frequently. We did not find any other significant difference in clinical or laboratory parameters between the two groups.

### 3.3. Treatment Modalities for LN and Non-LN Patients

When comparing the treatment of SLE, we found that corticosteroids, azathioprine, cyclophosphamide, mycophenolate mofetil, and rituximab were used significantly more frequently in LN patients. Conversely, the use of chloroquine and methotrexate was significantly more common in non-LN patients. The percentages of LLDAS patients and remission rates did not differ between the patient groups (Table 3).

### 3.4. Survival Rates and Causes of Death

**Figure 1:**
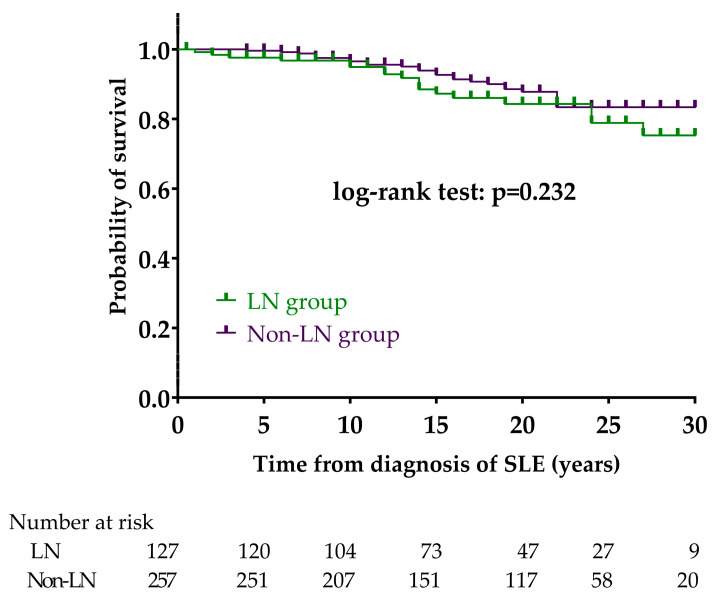
Kaplan–Meier analysis to compare survival between LN and non-LN groups. Abbreviations: SLE, systemic lupus erythematosus; LN, lupus nephritis; non-LN, SLE patients without lupus nephritis.

We compared the survival of LN and non-LN patients (Figure 1).

The 5-, 10-, 15-, 20-, 25- and 30-year survival rates in the LN group were 97.6%, 94.9%, 87.3%, 84.3%, 78.9%, and 75.3%, respectively. The 5-, 10-, 15-, 20-, 25- and 30-year survival rates in the non-LN group were 99.6%, 96.6%, 92.7%, 87.8%, 83.4%, and 83.4%, respectively. The mean survival time was 28.2 years (95% CI 26.6–29.8) in the LN group and 29.3 years (95% CI 28.4–30.3) in the non-LN group. We did not find a significant difference in survival between the two groups (*p* = 0.232).

Forty-five deaths (11.7%) occurred in the entire patient group. A total of 13 (28.9%) patients died of cardiovascular events and 12 (26.7%) due to some kind of infection. We lost 11 (24.4%) patients due to malignancy and 6 (13.3%) due to severe fulminant sepsis, while 3 (6.7%) died of other causes. There were 18 deaths in the LN group, mainly due to cardiovascular events (*n* = 5, 27.8%) and sepsis (*n* = 5, 27.8%), the latter was significantly more common in this group compared to the non-LN group. We observed 27 cases of death in the non-LN group; the most common causes were cardiovascular events (*n* = 8, 29.6%), and malignancies (*n* = 8, 29.6%) (Table 4).

**Figure 2:**
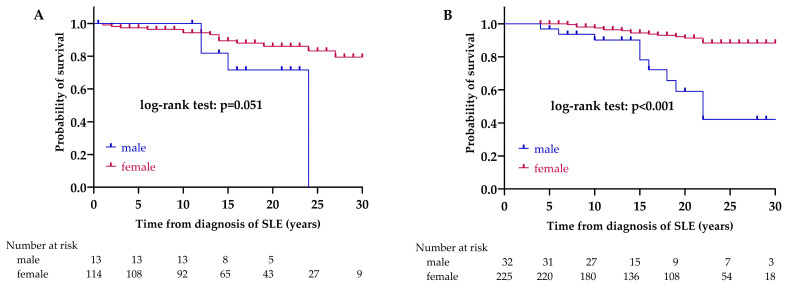
Effect of sex on survival in LN (**A**) and non-LN (**B**) group. Abbreviations: SLE, systemic lupus erythematosus; LN, lupus nephritis; non-LN, SLE patients without lupus nephritis.

We looked for factors that could determine the survival rates of patients. In the LN group, there was no significant difference between the sexes in survival (*p* = 0.051). There were 14/114 (12.3%) deaths among women and 4/13 (30.8%) among men. In the non-LN group, a significant difference was observed between the survival of women and men (*p* < 0.001). There were 17/225 (7.6%) deaths among women and 10/32 (30.8%) among men. Based on the result from the univariate Cox regression, the prognosis of men in the non-LN group is worse, compared to women; the hazard ratio (HR) is 5.43 (95% CI 2.47–11.92, *p* < 0.001) (Figure 2).

In the LN group, there was no significant difference between the age at SLE onset groups in survival (*p* = 0.454). There were 1/16 (6.3%) deaths among the <18 years group, 17/109 (15.6%) deaths among the 18–50 years group, and 0/2 (0%) deaths among >50 years group. In the non-LN group, there was no significant difference between the age at SLE onset groups in survival (*p* = 0.920). There were 2/15 (13.3%) deaths among the <18 years group, 23/216 (10.6%) deaths among the 18–50 years group, and 2/26 (7.7%) deaths among the >50 years group.

**Figure 3:**
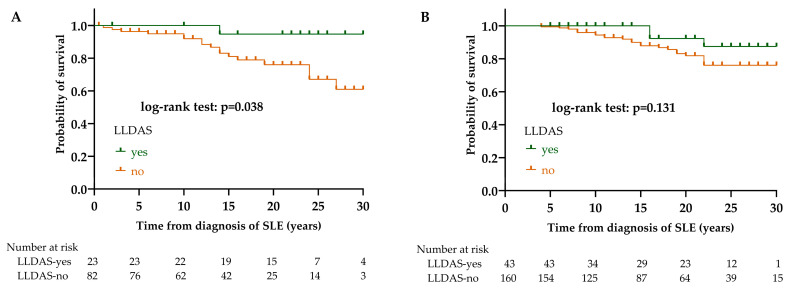
Effect of LLDAS on survival in LN (**A**) and non-LN (**B**) group. Abbreviations: LLDAS, lupus low disease activity state; SLE, systemic lupus erythematosus; LN, lupus nephritis; non-LN, SLE patients without lupus nephritis.

Regarding the effect of achieving lupus low disease activity state (LLDAS) on survival, we found that patients with LLDAS had significantly better survival in the LN group (*p* = 0.038). Death in patients who achieved LLDAS was 1/23 (4.3%), while in patients who did not achieve LLDAS, it was 17/82 (20.7%). In the non-LN group, LLDAS did not significantly affect survival (*p* = 0.131). Death in patients who achieved LLDAS was 3/43 (7.0%), while in patients who did not achieve LLDAS, it was 24/160 (15.0%) (Figure 3).

**Figure 4:**
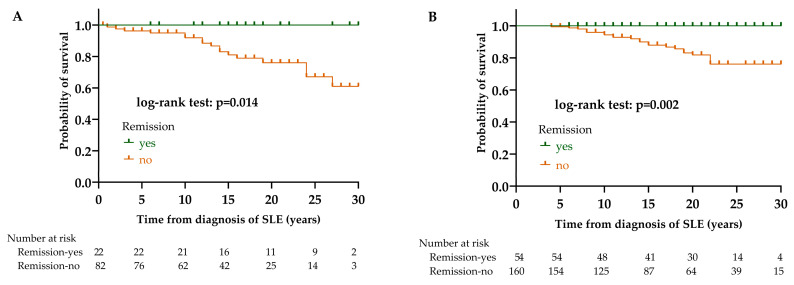
Effect of remission on survival in LN (**A**) and non-LN (**B**) group. Abbreviations: SLE, systemic lupus erythematosus; LN, lupus nephritis; non-LN, SLE patients without lupus nephritis.

The survival of patients in remission was significantly better in both patient groups (LN: *p* = 0.014; non-LN: *p* = 0.002). In the case of patients who did not achieve remission, there were 17/82 (20.7%) deaths in the LN group, while 24/160 (15.0%) in the non-LN group. In patients who achieved clinical or complete remission, there were no deaths in either group (Figure 4).

**Figure 5:**
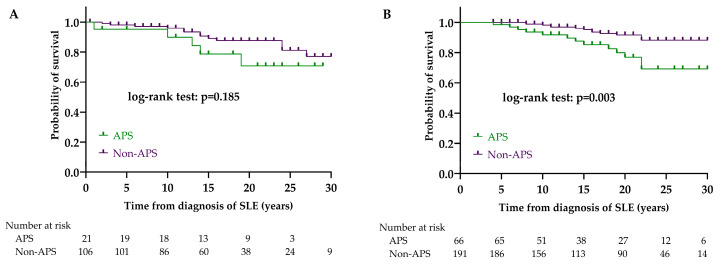
Effect of APS on survival in LN (**A**) and non-LN (**B**) group. Abbreviations: APS, antiphospholipid syndrome; SLE, systemic lupus erythematosus; LN, lupus nephritis; non-LN, SLE patients without lupus nephritis.

Regarding the clinical manifestations, we found no significant difference in the LN group between the patients with or without APS in survival (*p* = 0.185). There were 5/21 (23.8%) deaths among patients with APS and 13/106 (12.3%) among patients without APS. In the non-LN group, a significant difference was observed between the survival of patients with and without APS (*p* = 0.003). There were 13/66 (19.7%) deaths among patients with APS and 14/191 (7.3%) among patients without APS (Figure 5).

In the LN group, there was no significant difference between the patients with or without CNS manifestations in survival (*p* = 0.583). There were 5/25 (20%) deaths among patients with CNS manifestations and 13/102 (12.7%) among patients without CNS manifestations. In the non-LN group, there was no significant difference between the survival of patients with and without CNS manifestations (*p* = 0.883). There were 9/67 (13.4%) deaths among patients with CNS manifestations and 18/190 (9.5%) among patients without CNS manifestations.

**Figure 6:**
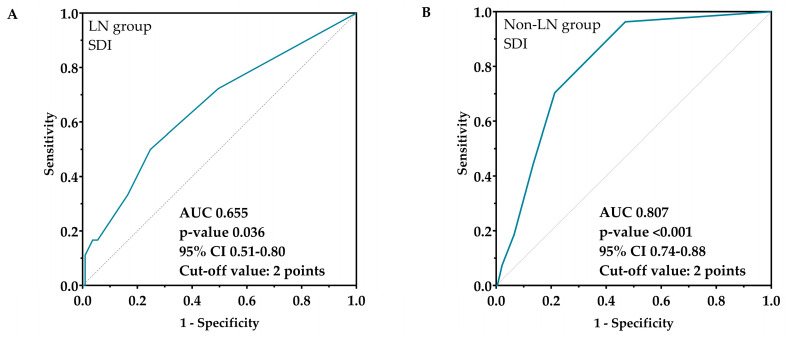
ROC curve and analysis of cut-off value of SDI as a prognostic factor for mortality in LN (**A**) and non-LN (**B**) group. Solid blue line: ROC curve; dotted line: chance diagonal (AUC 0.5). Based on the ROC analysis, the SDI’s optimal cut-off value was 2 points in both groups. Abbreviations: ROC, receiver operating characteristic analysis; LN, lupus nephritis; non-LN, SLE patients without lupus nephritis; AUC, area under the curve; 95% CI, 95% confidence interval.

We also examined the SDI’s impact on survival. Based on the ROC analysis, the SDI’s optimal cut-off value was 2 points in both groups; the area under the curve (AUC) in the LN group was 0.655 (*p* = 0.036, 95% CI 0.51–0.80, cut-off value: 2 points), while 0.807 in the non-LN group (*p* < 0.001, 95% CI 0.74–0.88, cut-off value: 2 points) (Figure 6).

**Figure 7:**
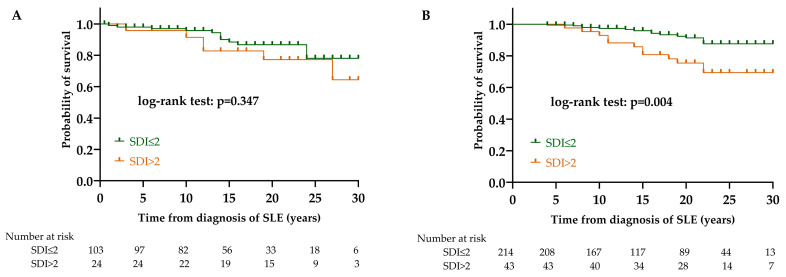
Effect of SDI on survival in LN (**A**) and non-LN (**B**) group. Abbreviations: SLE, systemic lupus erythematosus; SDI, SLICC/ACR Damage Index; LN, lupus nephritis; non-LN, SLE patients without lupus nephritis.

SDI did not affect the survival in the LN group (*p* = 0.347). Conversely, in the non-LN group, SDI had a significant effect on survival (*p* = 0.004). Based on the result from univariate Cox regression, the survival of those with more than 2 points in the non-LN group was significantly worse compared to those with fewer points (HR = 2.93 [95% CI 1.37–6.28]; *p* = 0.006) (Figure 7).

### 3.5. Differences between LN Patients Diagnosed before 2005 or since 2005

In the second part of the research, we were interested in whether certain histological types of LN, the clinical characteristics of patients, or the shad changed. Therefore, we divided LN patients into two groups and compared the aforementioned data of LN patients diagnosed before and since 2005. The follow-up data of patients diagnosed before 2005 were then only taken into account until 2005 so that the comparison between the two patient groups would be proportional. We observed no difference in the frequency of certain histological types before and after 2005 in our LN cohort (Table 5).

Based on our results, the prevalence of many clinical manifestations of SLE has decreased in the last 15 years compared to before. Raynaud’s phenomenon, DLE, pleuritis, pericarditis, central nervous system, and psychiatric manifestations as well as stroke became significantly less prevalent. The frequencies of chronic kidney disease and end-stage renal disease also showed a decreasing trend, although the differences were not significant. The SDI of patients improved significantly in the past 15 years. There have also been changes regarding the prescribed medicines. The use of cyclophosphamide significantly decreased, while the use of mycophenolate mofetil, chloroquine, and rituximab significantly increased. At the same time, the cumulative corticosteroid dose was significantly reduced (Table 6).

**Figure 8:**
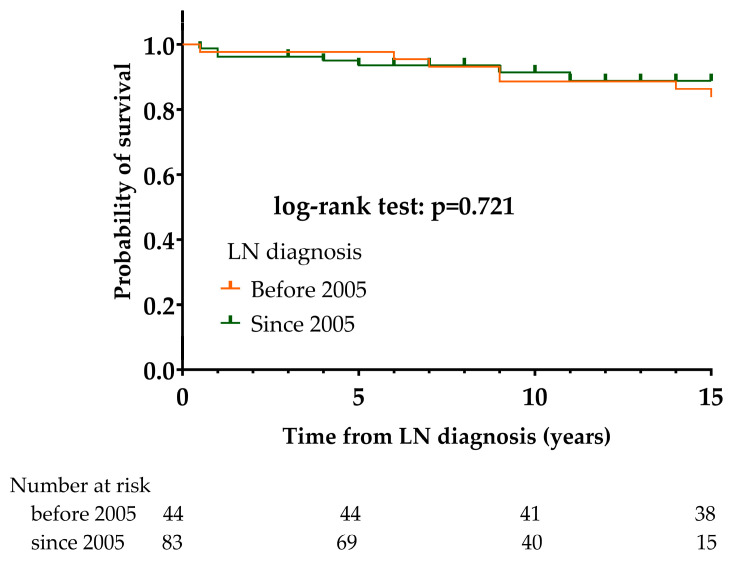
Comparison of mortality of LN patients diagnosed before and since 2005 using Kaplan–Meier survival estimates. Abbreviations: LN, lupus nephritis: SLE, systemic lupus erythematosus.

The 5-, 10-, and 15-year survival rates in the group of LN patients diagnosed before 2005 were 97.7%, 88.6%, and 84.1%, respectively; 7/44 (15.9%) patients died during the period of observation. The 5-, 10-, and 15-year survival rates among LN patients diagnosed since 2005 group were 93.6%, 91.5%, and 88.8%, respectively; 7/83 patients (8.4%) died during the period of observation. We did not find a significant difference in survival between the two groups (*p* = 0.721) (Figure 8).

## 4. Discussion

Lupus nephritis is a clinical manifestation that fundamentally influences the prognosis of SLE. LN can develop at any time during the course of the disease; nevertheless, its development most often occurs in the first 3–5 years of SLE, and sometimes LN can be one of the first symptoms of lupus [1]. The prevalence and severity of LN show geographic and ethnic variety. It may vary within the white population, as Greek researchers found an LN prevalence of 20.3% among 555 SLE patients, while the Spanish RELESSER registry with more than 4000 SLE patients’ data showed an LN prevalence similar to our results, in which almost one-third of SLE patients were affected [41,42]. Studies from other countries of the world showed an even higher LN prevalence. An Iranian study with a large number of SLE patients revealed a prevalence of 68.1%, Chinese authors reported 49–58% prevalence, while Columbian research found a 51% prevalence [43,44,45]. In most studies, the group of lupus patients with LN was significantly younger both at the time of diagnosis and at the time of data collection [45].

We compared the clinical characteristics of SLE patients between LN and non-LN patient groups. We found that the prevalence of SCLE and DLE are higher in the non-LN group, in which other systemic autoimmune diseases are also more common, such as secondary APS, Sjögren’s syndrome, and rheumatoid arthritis. Among our patients with lupus nephritis, only anemia was observed to be more common; hematological manifestations also occurred more frequently in Spanish LN patients. The Iranian workgroup found photosensitivity, malar rash, and central nervous system symptoms to be more common among LN patients, while polyarthritis and autoimmune hemolytic anemia occurred mostly among non-lupus-nephritis patients [43]. Based on data from the Spanish registry, serositis, skin symptoms, neurological manifestations, pulmonary involvement, and hematological diseases are associated more commonly with LN [44]. We could not confirm these findings, and contrary to the Spanish data, the association of APS was more common in non-LN Hungarian patients. Colombian authors also reported a higher prevalence of hypertension and pleurisy in LN patients; they also found anti-DNA positivity to be more common [45]. It is known that the presence of several autoantibodies can be associated with the occurrence of lupus nephritis. The association of anti-Sm, anti-nucleosome, or anti-C1q antibodies with lupus nephritis is well known [46]. We found anti-RNP to be significantly more common among patients with lupus nephritis; conversely, based on data from the RELESSER registry, anti-Sm is more common, while the Iranian working group found ANA positivity to be more common in their patients with LN [42,43]. We found no difference in the prevalence of ANA, anti-dsDNA, or anti-Sm antibodies. Since the measurement of anti-nucleosome and anti-C1q antibodies was not performed in all of our patients, we did not evaluate their presence. There is a working group that has associated anti-SSA with worse renal outcomes, The literature is conflicting regarding the presence of anti-SS-A. Some authors associated anti-SS-A with a worse renal outcome; while, conversely, the Colombian authors reported a protective role of anti-SS-A. We found no difference in the presence of anti-SS-A between our two patient groups [43,47]. Of note, all patients included in our retrospective study were white Europeans from the Central European region. The comparison of patients living in other countries or even other continents is significantly influenced by different environmental factors and the genetic background of the patients, which may explain the differences between our observations and the results of others.

As expected, LN patients were treated with cyclophosphamide, MMF, or rituximab more often, while antimalarial drugs were given less often. This can be explained by the fact that previously it was not the practice for all patients to receive antimalarial treatment. At the same time, when we examined the therapeutic changes of the last 15 years, the use of chloroquine in LN patients increased significantly. Similar changes occurred with other immunosuppressants, MMF is more frequently used as both induction and maintenance therapy. It is known that the effectiveness of cyclophosphamide and MMF is the same, but the side effect profile of MMF is more favorable, thus we prefer it in the case of young patients of fertile age; therefore, the use of MMF has increased, while the use of cyclophosphamide has decreased [13,17,48,49]. We observed another favorable therapeutic change: the cumulative corticosteroid dose has significantly reduced, which has a significant role in the reduction of chronic organ damage [29].

According to our results, the clinical picture of lupus nephritis has also changed. Despite the fact that the investigated clinical manifestations of SLE were not more common among LN patients compared to non-LN patients, there were changes in the prevalence of several organ abnormalities over the last 15 years. The prevalence of serositis and neuropsychiatric manifestations decreased significantly, as did the SDI. Recently, Mok and his colleagues compared data from two 10-year periods. They found that patients diagnosed in the second 10-year period had anti-ENA antibodies more frequently, which is probably attributable to the developing laboratory methodology; we did not find a similar difference [44]. They also showed, like us, that the SDI score also decreased significantly, which has an important impact on long-term survival. Based on our results, the SDI had an effect on the survival of non-LN patients: a damage score above 2 points significantly worsened their survival, while it did not affect the survival of LN patients. An Italian working group examined the clinical and histological changes in LN and the disease outcome in nearly 500 patients over a period of 50 years [50]. They concluded that the severity of lupus nephritis has decreased in recent years, and the outcome of the disease has also improved, which findings are confirmed by our results.

Examining survival, we found no significant difference in the short- and long-term survival of LN and non-LN patients. At the same time, our results underlined that the frequency of APS in the non-LN group is significantly higher; moreover, the presence of APS significantly impairs the survival of non-LN patients. Taken together, it can be assumed that the negative effect of LN on survival in the LN patient group may be comparable to the effect of APS on mortality in the non-LN group. Male sex did not significantly worsen survival among LN patients, while it proved to be a negative prognostic factor in non-LN patients. When examining the survival of SLE patients, Doria et al. identified male sex, the presence of lupus anticoagulant, and severe disease as negative prognostic factors, and found that glomerulonephritis also worsened survival rates [51]. Conversely, Lou et al. found no difference between men and women in either mortality or development of LN [52]. We also examined how achieving low disease activity and remission affects patients’ survival rates. It became clear that the achievement of remission significantly improves survival in both the LN and non-LN groups, while low disease activity is protective only in the lupus nephritis group. Based on the results of a prospective multicenter study, LLDAS significantly improved patient survival and remission did not add to it [53]. Our results showed that low disease activity is sufficient in patients with lupus nephritis, but in the non-LN group, the therapeutic goal should be to achieve remission. Achieving LLDAS and remission reduces the development of chronic damage and reduces SDI, thus contributing to improved survival [28]. The leading causes of death for our patients correspond to international data, according to which cardiovascular, infections, and tumors are the main causes of death [30,50]. Cardiovascular diseases are the leading cause of death in both lupus nephritis and non-LN patients. The occurrence of severe sepsis as a cause of death was significantly more frequent in the LN group, which is presumably explained by the more potent immunosuppressant treatment.

This study has some limitations. It was a retrospective study and some risk factors, were not available and could not be included in the analysis as covariates or potential confounders. Limitations also exist on the treatment adherence of the enrolled patients. Despite knowing the medications and the doses used and the proper accuracy of the administrative staff on the documentation of drug prescriptions, we cannot exclude the possibility of erratic patient adherence. However, with the methods of analysis used, none of these issues are likely to cause any systematic error or bias. Despite these limitations, our study had two major strengths. One is the length of the period examined, which was more than three decades in some cases, which can be considered good for observational studies. Another strength is that we investigated and analyzed multiple laboratory and clinical parameters as well as disease outcomes, providing more real-world data about the natural course of LN.

In conclusion, we found that DLE, SCLE, secondary APS, Sjögren’s syndrome, and rheumatoid arthritis were more common in non-LN SLE patients. Regarding laboratory parameters, anti-RNP positivity was significantly more common in our LN patients. There was no difference in the survival of LN and non-LN patients. Achieving LLDAS and remission improves survival rates in patients with LN. Among patients with LN diagnosed after 2005, the development of serositis and neurological manifestations was less common, and the SDI scores were significantly lower. The prevalence of CKD and ESRD did not show a significant decrease in LN patients diagnosed after 2005; however, an improvement has begun, and further improvement is expected due to the new innovative drugs. The main therapeutic goal in SLE should be to achieve remission, regardless of the organ manifestation, because this significantly improves the long-term prognosis and the survival of patients.

## Figures and Tables

**Table 1:** Demographic characteristics of SLE patients with and without LN.

	LN (*n* = 127)	Non-LN (*n* = 257)	*p*-Value
Sex (female)	114 (89.8)	225 (87.5)	0.525
Age, years	45.3 ± 11.6	53.5 ± 13.4	**<0.001**
Age onset SLE, years	28.4 ± 9.9	35.7 ± 12.1	**<0.001**
Duration of disease, years	16.9 ± 8.1	17.8 ± 8	0.319

Values are presented as number (%) and mean ± SD, *p*-values are calculated by Pearson’s chi-squared test and Student’s *t*-test for two samples. Bold indicates statistically significant (*p* < 0.05) result. Abbreviations: SLE, systemic lupus erythematosus; *n*, number of patients; LN, lupus nephritis; non-LN, SLE patients without lupus nephritis.

**Table 2:** Comparison of clinical manifestations and positive laboratory findings between patients with and without LN.

	LN (*n* = 127)	Non-LN (*n* = 257)	*p*-Value
Clinical manifestations			
*Mucocutan*			
Livedo reticularis	11 (8.7)	19 (7.4)	0.663
Acute skin lesions	53 (41.7)	93 (36.2)	0.292
DLE	6 (4.7)	47 (18.3)	**<0.001**
SCLE	7 (5.5)	37 (14.4)	**0.010**
Alopecia	38 (29.9)	59 (23)	0.140
Photosensitivity	29 (22.8)	79 (30.7)	0.105
Mucous ulcer	16 (12.6)	20 (7.8)	0.128
*Serositis*			
Pleuritis	32 (25.2)	63 (24.5)	0.884
Pericarditis	25 (19.7)	43 (16.7)	0.476
*Neuropsychiatric*			
CNS manifestations	25 (19.7)	67 (26.1)	0.168
PNS manifestations	10 (7.9)	30 (11.7)	0.252
Psychiatric manifestations	25 (19.7)	45 (17.5)	0.603
*Cardiovascular*			
APS	21 (16.5)	66 (25.7)	**0.044**
DVT	17 (13.4)	56 (21.8)	**0.048**
PE	4 (3.1)	11 (4.3)	0.781
AMI	3 (2.4)	3 (1.2)	0.402
Stroke	8 (6.3)	24 (9.3)	0.311
Obliterative arteriopathy	1 (0.8)	1 (0.4)	0.553
Valvulopathy	25 (19.7)	56 (21.8)	0.634
*Others*			
Polyarthritis	111 (87.4)	209 (81.3)	0.133
Spontaneous abortion (*n* = 339)	20 (17.5)	36 (16)	0.718
*Associated autoimmune diseases*			
RA	13 (10.2)	54 (21)	**0.009**
SS	5 (3.9)	29 (11.3)	**0.017**
PSS	2 (1.6)	0 (0)	0.109
SDI	1 (0–2)	1 (0–2)	0.782
Laboratory findings			
Thrombocytopenia	58 (45.7)	92 (35.8)	0.062
Leukopaenia	97 (76.4)	175 (68.1)	0.093
Anaemia	110 (86.6)	172 (66.9)	**<0.001**
Anti ß2-GP-1	67 (52.8)	127 (49.4)	0.538
Anti-CL	83 (65.4)	152 (59.1)	0.240
LA	23 (18.1)	54 (21)	0.504
ANA	113 (89)	212 (82.5)	0.097
Anti-dsDNA	120 (94.5)	228 (88.7)	0.068
Anti-Sm	52 (40.9)	85 (33.1)	0.130
Anti-RNP	46 (36.2)	68 (26.5)	**0.049**
Anti-SS-A (Ro)	77 (60.6)	168 (65.4)	0.363
Anti-SS-B (La)	51 (40.2)	119 (46.3)	0.254
ANCA	17 (13.4)	25 (9.7)	0.280
Cryoglobulin	7 (5.5)	6 (2.3)	0.134
Coombs test positivity	20 (15.7)	25 (9.7)	0.084

Values are presented as number (%), *p*-values are calculated by Pearson’s chi-squared and Fisher’s exact test. Bold indicates statistically significant (*p* < 0.05) result. Abbreviations: *n*, number of patients; LN, lupus nephritis; non-LN, SLE patients without lupus nephritis; DLE, discoid lupus erythematosus; SCLE, subacute cutaneous lupus erythematosus; CNS, central nervous system; PNS, peripheral nervous system; APS, antiphospholipid syndrome; DVT, deep vein thrombosis; PE, pulmonary embolism; AMI, acute myocardial infarction; RA, rheumatoid arthritis; SS, Sjögren’s syndrome; PSS, progressive systemic sclerosis; SDI, SLICC/ACR Damage Index; anti-ß2GPI, anti-β2-glycoprotein-1 antibody; anti-CL, anticardiolipin antibody; LA, lupus anticoagulant; ANA, antinuclear antibodies; anti-dsDNA, anti-double-stranded DNA; anti-Sm, anti-Smith antibody; anti-RNP, anti-ribonucleoprotein antibody; anti-SS-A, anti-Sjögren’s-syndrome-related antigen A autoantibody; anti-SS-B, anti-Sjögren’s-syndrome-related antigen B autoantibody; ANCA, antineutrophil cytoplasmic antibody.

**Table 3:** Comparison of the treatments and the treatment effects on patients with and without LN.

	LN (*n* = 127)	Non-LN (*n* = 257)	*p*-Value
Corticosteroid	127 (100)	239 (93)	**0.002**
Corticosteroid (currently)	81 (63.8)	148 (57.6)	0.245
CD of corticosteroid, mg/kg	16,060 (7300–32,120)	15,330 (5840–29,200)	0.255
Chloroquine	60 (47.2)	161 (62.6)	**0.004**
Azathioprine	96 (75.6)	107 (41.6)	**<0.001**
Mycophenolate mofetil	52 (40.9)	7 (2.7)	**<0.001**
Cyclophosphamide	103 (81.1)	53 (20.6)	**<0.001**
Methotrexate	12 (9.4)	60 (23.3)	**0.001**
Cyclosporin A	13 (10.2)	35 (13.6)	0.346
Plasmapheresis	26 (20.5)	35 (13.6)	0.084
Rituximab	18 (14.2)	9 (3.5)	**<0.001**
LLDAS	23 (18.1)	43 (16.7)	0.736
Remission	22 (17.3)	54 (21)	0.393

Values are presented as number (%) and median with IQR, *p*-values are calculated by Pearson’s chi-squared or and Mann–Whitney U. Bold indicates statistically significant (*p* < 0.05) result. Abbreviations: *n*, number of patients; LN, lupus nephritis; non-LN, SLE patients without lupus nephritis; CD, cumulative dose; LLDAS, lupus low disease activity state.

**Table 4:** Comparison of causes of death between patients with and without LN.

	LN (*n* = 18)	Non-LN (*n* = 27)	*p*-Value
CV	5 (27.8)	8 (29.6)	0.893
Infection	3 (16.7)	9 (33.3)	0.308
Sepsis	5 (27.8)	1 (3.7)	**0.031**
Tumor	3 (16.7)	8 (29.6)	0.482
Other ^a^	2 (11.1)	1 (3.7)	0.562

Values are presented as number (%), *p*-values are calculated by Pearson’s chi-squared and Fisher’s exact test. Bold indicates statistically significant (*p* < 0.05) result. ^a^ Other causes of death included suicide (*n* = 1) and underlying disease (*n* = 2). Abbreviations: *n*, number of patients; LN, lupus nephritis; non-LN, SLE patients without lupus nephritis; CV, cardiovascular.

**Table 5:** Kidney biopsy findings between patients of LN group (*n* = 127) diagnosed with SLE before (*n* = 44) and since (*n* = 83) 2005.

	LN (*n* = 127)	Before 2005 (*n* = 44)	Since 2005 (*n* = 83)	*p*-Value
Class I-II	7 (5.5)	4 (9.1)	3 (3.6)	0.234
Class III	13 (10.2)	4 (9.1)	9 (10.8)	1.000
Class IV	74 (58.3)	26 (59.1)	48 (57.8)	0.891
Class V	20 (15.7)	7 (15.9)	13 (15.7)	0.971
Mixed class	11 (8.7)	2 (4.5)	9 (10.8)	0.327
Class I–II + Class III	1 (9.1)	1 (50)	0 (0)	
Class I–II + Class IV	1 (9.1)	0 (0)	1 (11.1)	
Class I–II + Class V	1 (9.1)	0 (0)	1 (11.1)	
Class III + Class IV	2 (18.2)	1 (50)	1 (11.1)	
Class III + Class V	5 (45.5)	0 (0)	5 (55.6)	
Class IV + Class V	1 (9.1)	0 (0)	1 (11.1)	
Not classified	2 (1.6)	1 (2.3)	1 (1.2)	1.000

Values are presented as number (%), *p*-values are calculated by Pearson’s chi-squared test. Abbreviations: *n*, number of patients; LN, lupus nephritis.

**Table 6:** Changes in clinical manifestations and treatments between patients of LN group diagnosed with SLE before and since 2005.

	Before 2005 (*n* = 44)	Since 2005 (*n* = 83)	*p*-Value
Clinical manifestations			
*Mucocutan*			
Livedo reticularis	4 (9.1)	7 (8.4)	1.000
Acute skin lesions	21 (47.7)	32 (38.6)	0.319
DLE	5 (11.4)	1 (1.2)	**0.019**
SCLE	3 (6.8)	4 (4.8)	0.693
Alopecia	14 (31.8)	24 (28.9)	0.734
Photosensitivity	10 (22.7)	19 (22.9)	0.983
Mucous ulcer	4 (9.1)	12 (14.5)	0.386
*Serositis*			
Pleuritis	17 (38.6)	15 (18.1)	**0.011**
Pericarditis	15 (34.1)	10 (12)	**0.003**
*Neuropsychiatric*			
CNS manifestations	14 (31.8)	11 (13.3)	**0.012**
PNS manifestations	4 (9.1)	6 (7.2)	0.737
Psychiatric manifestations	13 (29.5)	12 (14.5)	**0.042**
*Cardiovascular*			
APS	9 (20.5)	12 (14.5)	0.387
DVT	5 (11.4)	12 (14.5)	0.626
PE	1 (2.3)	3 (3.6)	1.000
AMI	2 (4.5)	1 (1.2)	0.275
Stroke	6 (13.6)	2 (2.4)	**0.020**
Obliterative arteriopathy	0 (0)	1 (1.2)	1.000
Valvulopathy	9 (20.5)	16 (19.3)	0.874
*Renal*			
CKD	15 (34.1)	20 (24.1)	0.230
ESRD	6 (13.6)	6 (7.2)	0.339
Others			
Raynaud-syndrome	21 (47.7)	24 (28.9)	**0.035**
Polyarthritis	41 (93.2)	70 (84.3)	0.153
Spontaneous abortion (*n* = 114)	7 (17.9)	13 (17.3)	0.935
*Associated autoimmune diseases*			
RA	4 (9.1)	9 (16.9)	1.000
SS	0 (0)	5 (6)	0.163
PSS	0 (0)	2 (2.4)	0.544
SDI	1 (0–3)	0 (0–1)	**0.001**
Treatments			
Corticosteroid (currently)	26 (59.1)	55 (66.3)	0.423
CD of corticosteroid, mg/kg	26,280 (18,250–42,340)	11,680 (5840–24,090)	**<0.001**
Chloroquine	12 (27.3)	48 (57.8)	**0.001**
Azathioprine	36 (81.8)	60 (72.3)	0.234
Maintenance Therapy	33 (75)	48 (57.8)	0.055
Mycophenolate mofetil			
Induction Therapy	5 (11.4)	47 (56.6)	**<0.001**
Maintenance Therapy	2 (4.5)	33 (39.8)	**<0.001**
Cyclophosphamide	40 (90.9)	63 (75.9)	**0.040**
Methotrexate	5 (11.4)	7 (8.4)	0.751
Cyclosporin A	6 (13.6)	7 (8.4)	0.751
Plasmapheresis	10 (22.7)	16 (19.3)	0.647
Rituximab	1 (2.3)	17 (20.5)	**0.005**
Belimumab	2 (4.5)	6 (7.2)	0.713

Values are presented as number (%) and median with IQR, *p*-values are calculated by Pearson’s chi-squared or Fisher’s exact test and Mann–Whitney U test. Bold indicates statistically significant (*p* < 0.05) result. Abbreviations: SLE, systemic lupus erythematosus; *n*, number of patients; DLE, discoid lupus erythematosus; SCLE, subacute cutaneous lupus; CNS, central nervous system; PNS, peripheral nervous system; APS, antiphospholipid syndrome; DVT, deep vein thrombosis; PE, pulmonary embolism; AMI, acute myocardial infarction; CKD, chronic kidney disease; ESRD, end-stage renal disease; RA, rheumatoid arthritis; SS, Sjögren’s syndrome; PSS, progressive systemic sclerosis; SDI, SLICC/ACR Damage Index; CD, cumulative dose.

## Data Availability

The original contributions presented in the study are included in the article, further inquiries can be directed to the corresponding author/s.

## References

[1] Anders H.J., Saxena R., Zhao M.H., Parodis I., Salmon J.E., Mohan C. (2020). Lupus nephritis. Nat. Rev. Dis. Primers.

[2] Rovin B.H., Stillman I.K., Lahita R. (2011). Systemic Lupus Erythematosus.

[3] Hanly J.G., O’Keeffe A.G., Su L., Urowitz M.B., Romero-Diaz J., Gordon C., Bae S.C., Bernatsky S., Clarke A.E., Wallace D.J. (2016). The frequency and outcome of lupus nephritis: Results from an international inception cohort study. Rheumatology.

[4] Yurkovich M., Vostretsova K., Chen W., Aviña-Zubieta J.A. (2014). Overall and cause-specific mortality in patients with systemic lupus erythematosus: A meta-analysis of observational studies. Arthritis Care Res..

[5] Lee Y.H., Choi S.J., Ji J.D., Song G.G. (2016). Overall and cause-specific mortality in systemic lupus erythematosus: An updated meta-analysis. Lupus.

[6] Garcia M.A., Marcos J.C., Marcos A.I., Pons-Estel B.A., Wojdyla D., Arturi A., Babini J.C., Catoggio L.J., Alarcon-Segovia D. (2005). Male systemic lupus erythematosus in a Latin-American inception cohort of 1214 patients. Lupus.

[7] Andrade R.M., Alarcón G.S., Fernández M., Apte M., Vilá L.M., Reveille J.D., LUMINA Study Group (2007). Accelerated damage accrual among men with systemic lupus erythematosus: XLIV. Results from a multiethnic US cohort. Arthritis Rheum..

[8] Stefanidou S., Benos A., Galanopoulou V., Chatziyannis I., Kanakoudi F., Aslanidis S., Boura P., Sfetsios T., Settas L., Katsounaros M. (2011). Clinical expression and morbidity of systemic lupus erythematosus during a post-diagnostic 5-year follow-up: A male:female comparison. Lupus.

[9] Ramírez Gómez L.A., Uribe Uribe O., Osio Uribe O., Grisales Romero H., Cardiel M.H., Wojdyla D., Pons-Estel B.A., Catoggio L.J., Soriano E.R., Grupo Latinoamericano de Estudio del Lupus (GLADEL) (2008). Childhood systemic lupus erythematosus in Latin America. The GLADEL experience in 230 children. Lupus.

[10] Hoffman I.E., Lauwerys B.R., De Keyser F., Huizinga T.W., Isenberg D., Cebecauer L., Dehoorne J., Joos R., Hendrickx G., Houssiau F. (2009). Juvenile-onset systemic lupus erythematosus: Different clinical and serological pattern than adult-onset systemic lupus erythematosus. Ann. Rheum. Dis..

[11] Tarr T., Dérfalvi B., Győri N., Szántó A., Siminszky Z., Malik A., Szabó A.J., Szegedi G., Zeher M. (2015). Similarities and differences between pediatric and adult patients with systemic lupus erythematosus. Lupus.

[12] Gordon C., Jayne D., Pusey C., Adu D., Amoura Z., Aringer M., Ballerin J., Cervera R., Calvo-Alén J., Chizzolini C. (2009). European consensus statement on the terminology used in the management of lupus glomerulonephritis. Lupus.

[13] Bertsias G.K., Tektonidou M., Amoura Z., Aringer M., Bajema I., Berden J.H., Boletis J., Cervera R., Dörner T., Doria A. (2012). Joint European League Against Rheumatism and European Renal Association-European Dialysis and Transplant Association (EULAR/ERA-EDTA) recommendations for the management of adult and paediatric lupus nephritis. Ann. Rheum. Dis..

[14] Weening J.J., D’Agati V.D., Schwartz M.M., Seshan S.V., Alpers C.E., Appel G.B., Balow J.E., Bruijn J.A., Cook T., Ferrario F. (2004). International Society of Nephrology Working Group on the Classification of Lupus Nephritis; Renal Pathology Society Working Group on the Classification of Lupus Nephritis. The classification of glomerulonephritis in systemic lupus erythematosus revisited. Kidney Int..

[15] Bajema I.M., Wilhelmus S., Alpers C.E., Bruijn J.A., Colvin R.B., Cook H.T., D’Agati V.D., Ferrario F., Haas M., Jennette J.C. (2018). Revision of the International Society of Nephrology/Renal Pathology Society classification for lupus nephritis: Clarification of definitions, and modified National Institutes of Health activity and chronicity indices. Kidney Int..

[16] Kidney Disease: Improving Global Outcomes (KDIGO) Lupus Nephritis Work Group Collaborators (2024). KDIGO 2024 Clinical Practice Guideline for the management of Lupus Nephritis. Kidney Int..

[17] Fanouriakis A., Kostopoulou M., Andersen J., Aringer M., Arnaud L., Bae S.C., Boletis J., Bruce I.N., Cervera R., Doria A. (2024). EULAR recommendations for the management of systemic lupus erythematosus: 2023 update. Ann. Rheum. Dis..

[18] Furie R., Rovin B.H., Houssiau F., Malvar A., Teng Y.K.O., Contreras G., Amoura Z., Yu X., Mok C.C., Santiago M.B. (2020). Two-Year, Randomized, Controlled Trial of Belimumab in Lupus Nephritis. N. Engl. J. Med..

[19] Tanaka Y., Nakayamada S., Yamaoka K., Ohmura K., Yasuda S. (2023). Rituximab in the real-world treatment of lupus nephritis: A retrospective cohort study in Japan. Mod. Rheumatol..

[20] Rovin B.H., Teng Y.K.O., Ginzler E.M., Arriens C., Caster D.J., Romero-Diaz J., Gibson K., Kaplan J., Lisk L., Navarra S. (2021). Efficacy and safety of voclosporin versus placebo for lupus nephritis (AURORA 1): A double-blind, randomised, multicentre, placebo-controlled, phase 3 trial. Lancet.

[21] Furie R.A., Aroca G., Cascino M.D., Garg J.P., Rovin B.H., Alvarez A., Fragoso-Loyo H., Zuta-Santillan E., Schindler T., Brunetta P. (2022). B-cell depletion with obinutuzumab for the treatment of proliferative lupus nephritis: A randomised, double-blind, placebo-controlled trial. Ann. Rheum. Dis..

[22] Jayne D., Rovin B., Mysler E.F., Furie R.A., Houssiau F.A., Trasieva T., Knagenhjelm J., Schwetje E., Chia Y.L., Tummala R. (2022). Phase II randomised trial of type I interferon inhibitor anifrolumab in patients with active lupus nephritis. Ann. Rheum. Dis..

[23] Fanouriakis A., Kostopoulou M., Alunno A., Aringer M., Bajema I., Boletis J.N., Cervera R., Doria A., Gordon C., Govoni M. (2019). 2019 update of the EULAR recommendations for the management of systemic lupus erythematosus. Ann. Rheum. Dis..

[24] Morand E.F., Furie R., Tanaka Y., Bruce I.N., Askanase A.D., Richez C., Bae S.C., Brohawn P.Z., Pineda L., Berglind A. (2020). TULIP-2 Trial Investigators. Trial of Anifrolumab in Active Systemic Lupus Erythematosus. N. Engl. J. Med..

[25] van Vollenhoven R., Voskuyl A., Bertsias G., Aranow C., Aringer M., Arnaud L., Askanase A., Balážová P., Bonfa E., Bootsma H. (2017). A framework for remission in SLE: Consensus findings from a large international task force on definitions of remission in SLE (DORIS). Ann. Rheum. Dis..

[26] Golder V., Tsang-A-Sjoe M.W.P. (2020). Treatment targets in SLE: Remission and low disease activity state. Rheumatology.

[27] Petri M., Magder L.S. (2018). Comparison of Remission and Lupus Low Disease Activity State in Damage Prevention in a United States Systemic Lupus Erythematosus Cohort. Arthritis Rheumatol..

[28] Ugarte-Gil M.F., Wojdyla D., Pons-Estel G.J., Catoggio L.J., Drenkard C., Sarano J., Berbotto G.A., Borba E.F., Sato E.I., Tavares Brenol J.C. (2017). GLADEL. Remission and Low Disease Activity Status (LDAS) protect lupus patients from damage occurrence: Data from a multiethnic, multinational Latin American Lupus Cohort (GLADEL). Ann. Rheum. Dis..

[29] Tarr T., Papp G., Nagy N., Cserép E., Zeher M. (2017). Chronic high-dose glucocorticoid therapy triggers the development of chronic organ damage and worsens disease outcome in systemic lupus erythematosus. Clin. Rheumatol..

[30] Mok C.C., Kwok R.C., Yip P.S. (2013). Effect of renal disease on the standardized mortality ratio and life expectancy of patients with systemic lupus erythematosus. Arthritis Rheum..

[31] Tan E.M., Cohen A.S., Fries J.F., Masi A.T., McShane D.J., Rothfield N.F., Schaller J.G., Talal N., Winchester R.J. (1982). The 1982 revised criteria for the classification of systemic lupus erythematosus. Arthritis Rheum..

[32] Hochberg M.C. (1997). Updating the American College of Rheumatology revised criteria for the classification of systemic lupus erythematosus. Arthritis Rheum..

[33] Petri M., Orbai A.M., Alarcón G.S., Gordon C., Merrill J.T., Fortin P.R., Bruce I.N., Isenberg D., Wallace D.J., Nived O. (2012). Derivation and validation of the systemic lupus interna-tional collaborating clinics classification criteria for systemic lupus erythematosus. Arthritis Rheum..

[34] Aringer M., Costenbader K., Daikh D., Brinks R., Mosca M., Ramsey-Goldman R., Smolen J.S., Wofsy D., Boumpas D.T., Kamen D.L. (2019). 2019 European League against Rheumatism/American College of Rheumatology Classification Criteria for Systemic Lupus Erythematosus. Arthritis Rheumatol..

[35] Wilson W.A., Gharavi A.E., Koike T., Lockshin M.D., Branch D.W., Piette J.C., Brey R., Derksen R., Harris E.N., Hughes G.R. (1999). International consensus statement on preliminaryclassification criteria for definite an-tiphospholipid syndrome: Reportof an international workshop. Arthritis Rheum..

[36] Miyakis S., Lockshin M.D., Atsumi T., Branch D.W., Brey R.L., Cervera R., Derksen R.H., DE Groot P.G., Koike T., Meroni P.L. (2006). International consensus statement on an update of the classification criteria for definite antiphospholipid syndrome (APS). J. Thromb. Haemost..

[37] Barbhaiya M., Zuily S., Naden R., Hendry A., Manneville F., Amigo M.C., Amoura Z., Andrade D., Andreoli L., Artim-Esen B. (2023). ACR/EULAR APS Classification Criteria Collaborators. The 2023 ACR/EULAR Antiphospholipid Syndrome Classification Criteria. Arthritis Rheumatol..

[38] Churg J., Sobin L. (1982). Renal Disease: Classification and Atlas of Glomerular Disease.

[39] Cattran D.C., Feehally J., Cook H.T., Liu Z.H., Fervenza F.C., Mezzano S.A., Floege J., Nachman P.H., Gipson D.S., Praga M. (2012). Kidney disease: Improving global outcomes (KDIGO) glomerulonephritis work group. KDIGO clinical practice guideline for glomerulonephritis. Kidney Int. Suppl..

[40] Gladman D., Ginzler E., Goldsmith C., Fortin P., Liang M., Urowitz M., Bacon P., Bombardieri S., Hanly J., Hay E. (1996). The development and initial validation of the Systemic Lupus International Collaborating Clinics/American College of Rheumatology damage index for systemic lupus erythematosus. Arthritis Rheum..

[41] Nikolopoulos D., Kostopoulou M., Pieta A., Karageorgas T., Tseronis D., Chavatza K., Flouda S., Rapsomaniki P., Banos A., Kremasmenou E. (2020). Evolving phenotype of systemic lupus erythematosus in Caucasians: Low incidence of lupus nephritis, high burden of neuropsychiatric disease and increased rates of late-onset lupus in the ‘Attikon’ cohort. Lupus.

[42] Galindo-Izquierdo M., Rodriguez-Almaraz E., Pego-Reigosa J.M., López-Longo F.J., Calvo-Alén J., Olivé A., Fernández-Nebro A., Martinez-Taboada V., Vela-Casasempere P., Freire M. (2016). Characterization of Patients with Lupus Nephritis Included in a Large Cohort From the Spanish Society of Rheumatology Registry of Patients with Systemic Lupus Erythematosus (RELESSER). Medicine.

[43] Faezi S.T., Almodarresi M.H., Paragomi P., Gharibdoost F., Akhlaghi M., Jamshidi A., Shafaie N., Akbarian M. (2017). Clinical picture of lupus nephritis in patients with systemic lupus erythematosus (SLE): Results of a large survey. Rheum. Res..

[44] Mok C.C., Ho L.Y., Chan K.L., Tse S.M., To C.H. (2020). Trend of Survival of a Cohort of Chinese Patients With Systemic Lupus Erythematosus over 25 Years. Front. Med..

[45] Anaya J.M., Cañas C., Mantilla R.D., Pineda-Tamayo R., Tobón G.J., Herrera-Diaz C., Rendón D.M., Rojas-Villarraga A., Uribe M. (2011). Lupus nephritis in Colombians: Contrasts and comparisons with other populations. Clin. Rev. Allergy Immunol..

[46] Dema B., Charles N. (2016). Autoantibodies in SLE: Specificities, Isotypes and Receptors. Antibodies.

[47] Korbet S.M., Lewis E.J., Schwartz M.M., Reichlin M., Evans J., Rohde R.D. (2000). Factors predictive of outcome in severe lupus nephritis. Lupus Nephritis Collaborative Study Group. Am. J. Kidney Dis..

[48] Henderson L., Masson P., Craig J.C., Flanc R.S., Roberts M.A., Strippoli G.F., Webster A.C. (2012). Treatment for lupus nephritis. Cochrane Database Syst. Rev..

[49] Hahn B.H., McMahon M.A., Wilkinson A., Wallace W.D., Daikh D.I., Fitzgerald J.D., Karpouzas G.A., Merrill J.T., Wallace D.J., Yazdany J. (2012). American College of Rheumatology. American College of Rheumatology guidelines for screening, treatment, and management of lupus nephritis. Arthritis Care Res..

[50] Moroni G., Vercelloni P.G., Quaglini S., Gatto M., Gianfreda D., Sacchi L., Raffiotta F., Zen M., Costantini G., Urban M.L. (2018). Changing patterns in clinical-histological presentation and renal outcome over the last five decades in a cohort of 499 patients with lupus nephritis. Ann. Rheum. Dis..

[51] Doria A., Iaccarino L., Ghirardello A., Zampieri S., Arienti S., Sarzi-Puttini P., Atzeni F., Piccoli A., Todesco S. (2006). Long-term prognosis and causes of death in systemic lupus erythematosus. Am. J. Med..

[52] Luo W., Farinha F., Isenberg D.A., Rahman A. (2022). Survival analysis of mortality and development of lupus nephritis in patients with systemic lupus erythematosus up to 40 years of follow-up. Rheumatology.

[53] Kandane-Rathnayake R., Golder V., Louthrenoo W., Chen Y.H., Cho J., Lateef A., Hamijoyo L., Luo S.F., Wu Y.J., Navarra S.V. (2022). Asia-Pacific Lupus Collaboration. Lupus low disease activity state and remission and risk of mortality in patients with systemic lupus erythematosus: A prospective, multinational, longitudinal cohort study. Lancet Rheumatol..

